# Social and Poor Law Problems

**Published:** 1906-11-03

**Authors:** 


### SOCIAL AND POOR LAW PROBLEMS.

### LONDON AND PROVINCIAL PAUPERISM.

The Blue-book giving the returns of pauperism up to the beginning of this year contains satisfactory information in showing a decrease in the number of persons receiving relief, but as compared with the country as a whole London does not occupy a good position. The total number of paupers registered as receiving relief on January 1st of this year, exclusive of casuals and insane, was 802,068, of whom 122,555 belonged to London Unions. While the total number of persons relieved shows a slight decrease as compared with the year ending January 1st, 1905, the London district shows a slight increase, only 616 indeed, but regrettable in view of the fact that elsewhere in the country the number of paupers is smaller than it was. Moreover, while taking the country as a whole the proportion of paupers to population, as reckoned by the Registrar-General's estimates, was 27.1 per thousand, the proportion in London is 31.7 per thousand. This is the more noteworthy since elsewhere pauperism has shown a decrease in towns and industrial centres, while increasing in the distinctively rural neighbourhoods. This may be explained to some extent by the fact that London profits less than many other towns by an improvement in the state of a single trade, while it has the advantage of not suffering so severely from the slackness of any particular industry. Such towns as, for example, Manchester and Sheffield are more likely to vary between the extremes of prosperity and destitution than the Metropolis. But London lives largely by its own extension, and slackness in the building trade, which employs so much unskilled labour, always leads to an increase of pauperism. It is satisfactory, however, to see that in the southern district of London there is a decrease in the number of paupers, notably in the Wandsworth Union. The tendency of the year seems to be to offer indoor relief more generally, and in London as well as in other places this has been concurrent with a decrease in outdoor relief. It must be admitted that the present method of reckoning pauperism, by which the returns say only how many persons were in receipt of relief on a given day, does not give a perfectly accurate view of the poverty of the nation. It is quite possible that a large number of persons might be destitute in January who are self-supporting for the greater part of the year. It would be desirable to know how long a recipient needs parish help. A comparatively smaller number who need relief for a prolonged time means greater distress than a great many who are in need for a week or two; and the new returns asked for by the Local Government Board, which will give such details, should prove very valuable, and help to define the relations of poverty and pauperism which art not, so often as might be thought, convertible terms.

Messrs. Doulton and Company, Limited, have been awarded a Diploma of Honour and the Grand Prix at the Milan Exhibition in the Hygienic Section for sanitary appliances.

